# Understanding the human fit of access during initial recovery from the COVID-19 pandemic: a qualitative study in English general practice

**DOI:** 10.1136/bmjopen-2024-095120

**Published:** 2025-10-15

**Authors:** Jonathan Hammond, Rebecca Goulding, Jessica Drinkwater, Lindsey Kent, Simon Bailey, Kath Checkland, Jennifer Voorhees

**Affiliations:** 1 Division of Population Health, Health Services Research and Primary Care; School of Health Sciences, The University of Manchester, Manchester, UK; 2 Centre for Health Services Studies, University of Kent, Canterbury, Kent, UK

**Keywords:** COVID-19, Health Services Accessibility, Primary Care, Health policy

## Abstract

**Abstract:**

**Objectives:**

Access to general practice in England is a challenging issue of enduring importance. COVID-19 precipitated various abrupt changes, exposing and compounding existing problems. The access as human fit conceptualisation provides a nuanced understanding of access that extends beyond a limited focus on appointment numbers and speed. This qualitative study explored the pandemic’s impact on access to general practice and the experiences of patients and healthcare staff in England using access as human fit as an analytical framework.

**Design:**

A community-based participatory approach underpinned by qualitative semi-structured interviews and focus groups, and observations.

**Setting and participants:**

The following were conducted in Northwest England (December 2021—August 2022): interviews (10 participants) with patients, general practice staff and professionals; seven focus groups (42 participants) with patients from general practice patient groups and underserved groups; and twenty observation sessions of non-clinical access encounters (seven general practice and Primary Care Network premises; 45 hours total).

**Analysis:**

A rapid qualitative analysis methodology facilitated an abductive thematic approach, applying the dimensions of access as human fit to the data.

**Results:**

The access as human fit framework highlighted key areas where there is a lack of fit between patients and staff. Patients expressed that the array of access options and changes made it hard to know how to be a patient; some thought general practice should be ‘back to normal’ and the pandemic was an excuse to restrict access. Providers reported working harder than ever with insufficient resources.

**Conclusions:**

The pandemic created greater distance between staff and patient realities of access. Access as a human fit facilitated in-depth exploration of patient and staff experiences, improving understanding and identifying key issues. Broader adoption and application of this framework, within policy and practice, could focus improvement efforts, optimise access fit and improve patient satisfaction and staff retention.

**STRENGTHS AND LIMITATIONS OF THIS STUDY**

- This research addresses a persistent gap in the literature about nuanced understandings of experiences of access from patient and staff perspectives in the relevant context of recovery from the COVID-19 pandemic.
- This paper uses the application of access as human fit, a conceptualisation of access that accounts for the complexities of patient and staff interactions, to contextualise the findings within broader discussions of access in the literature.
- The study used an iterative combination of qualitative methods—interviews, focus groups and observations—to support understanding of the access experiences of a diverse range of participants, including those from underserved groups.
- The research applied a participatory approach, partnering with a Community-Based Research Team throughout to ensure perspectives of a group with diverse characteristics, roles and experiences were reflected in the decisions of the research.
- This paper focuses on one geographical area in England and inevitably reflects the experiences of a particular time during pandemic recovery.

## Introduction

 Access to primary healthcare has long been a crucial issue for patients and healthcare providers. Prior to the COVID-19 pandemic, general practice access in the English NHS was the subject of repeated top-down policy interventions attempting to increase the number of appointments and the speed at which patients could be seen, due to perceived patient dissatisfaction and associated political pressures.^1^ With the arrival of COVID-19 in March 2020, the need to protect the safety of patients and staff resulted in overnight changes to access arrangements, which accelerated existing trends^2^ toward ‘total triage’ and remote by default appointments.^3^ The pandemic represented an exogenous shock^4^ to general practice, which exposed and compounded problems of access to care in myriad ways in different contexts. These changes continue to ripple and evolve with significance for the experiences of people seeking care, general practice staff and the health of populations.^5^

**Figure 1:**
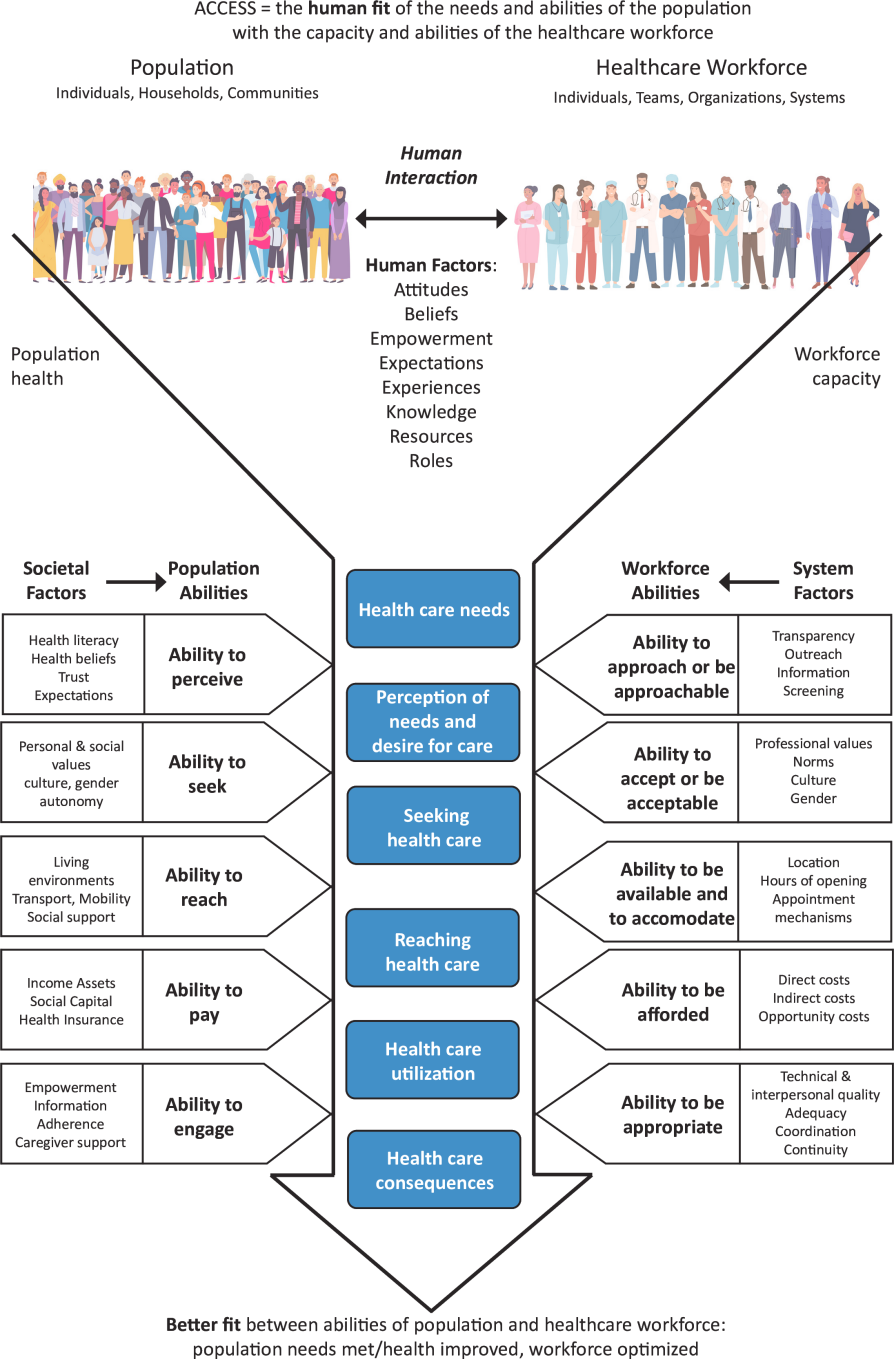
Access as human fit conceptualisation (reproduced from Voorhees et al, 2021, BJGP, https://doi.org/10.3399/BJGP.2021.0375, under Creative Commons Attribution License).

Despite historically simplistic policy definitions of access, which focus on timeliness,^6^ there is academic recognition that access is a dynamic question of the fit that can be achieved between healthcare systems and populations.^7^,^10^ Pre-pandemic, Voorhees *et al*,^11^ building on Levesque *et al*’s^12^ work, developed a conceptual framework of access to general practice highlighting the importance of human fit between patients’ needs and abilities and the capacity and abilities of the general practice workforce. Integral to this is an understanding that human factors affecting and shaping access are complex, that continuity is a fundamental element of access and that increasing the flexible delivery of care could help address access inequalities. Figure 1 demonstrates this as the access as human fit conceptualisation. In it, the population and healthcare workforce have the potential to connect across five dimensions to determine the human fit of access.

Whether and how these dimensions fit together represent people’s experiences of accessing and providing care. All dimensions are mediated in contextually specific and dynamic ways by broader societal factors for patients and by the system factors for staff (while also recognising that staff are patients and members of society too). By better connecting the dimensions at each stage of the healthcare access process, patients and staff can work together to move towards greater fit. While understanding that this process is frequently not linear,^13^ the application of this understanding of access complements existing theory around patient experience^14^ to refine improving access as improving human fit.

The aim of this paper is to explore the impact of the pandemic on general practice access in the English NHS, from the perspectives of patients and staff, during initial phases of recovery.

## Methods

### Study design and setting

This paper focuses on a qualitative work package of a larger 26-month mixed methods study (May 2021—June 2023), which took place in two local authority areas in Northwest England. It is important to set the context in which the data was collected. During the first UK ‘lockdown’ in March 2020, general practices were advised to shift to remote consultations unless there was an urgent need for a face-to-face appointment.^2^ In December 2021, national plan B measures were introduced in response to rapid transmission, including compulsory face coverings in certain public venues and work from home guidance. These were relaxed early in 2022, along with the duty to self-isolate. This phase of the pandemic saw the announcement of Living with COVID-19 guidance in England, with no further free universal symptomatic and asymptomatic public testing.^15^ This included an end to social distancing in general practices (as part of the ‘stepping down’ rules),^16^ but these changes took place in a context of increased and sustained demand for general practice services, workforce pressures and the ongoing involvement of many practices in vaccine delivery.^17^

### Patient and public involvement

The research project used a community-based participatory approach,^18 19^ characterised by close working with a Community-Based Research Team comprising general practitioners (GPs), patients, carers, voluntary sector providers, commissioners and other local stakeholders drawn from the local context where the study took place. This 12-member team, established during the preceding project,^11^ was actively involved in shaping the focus and approach of the current study and provided invaluable knowledge about the local health and care context. The team met 19 times during the research. Members informed decisions regarding the recruitment of general practice sites and supported the facilitation of focus groups, observations and data analysis. Members of the team have contributed to the dissemination of the findings from the study locally and nationally via events and educational sessions for the public and local general practice staff and a presentation at the Royal College for General Practitioners Annual Conference, which was co-presented.

### Participants and data collection

In-depth interviews (10) with patients, general practice staff and professionals (from a range of general practices with varied characteristics in terms of number of employees, patient population demographics and organisational processes relevant to access), including voluntary, community and social enterprise sector (VCSE) providers, and seven focus groups (42 participants) with different patients’ groups took place in a range of community settings (December 2021–August 2022). Focus groups and interviews were conducted by JH, RG, JV and JD, all experienced qualitative health services researchers. JV and JD are also clinical academics working in general practice. Patients were recruited for interviews and focus groups via patient participation groups and existing community groups, particularly those supporting marginalised or underserved groups that may experience access challenges. See table 1 for a breakdown of focus group and interview participants by community group/participant role and data collection method. General practice staff and professionals were recruited for interviews via pre-existing contacts, suggestions of members of the Community-Based Research Team (see Patient and Public Involvement section) and snowballing.

**Table 1:** Focus group and interview participants by data collection method

Participant type	Community group type/participant role	Data collection method	Number of participants	Number of sessions	Mode of data collection	Researcher(s) facilitating	Duration range (mins)
Patients							
	Mental health support group	Focus group/interview	8	4	In person	JH, RG	16–38
	BAME (Black, Asian and Minority Ethnic) community group	Focus group	11	2	In person	JH, RG	23–38
	Refugee and asylum seeker group	Focus group	15	1	In person	RG, JD	58
	Learning and developmental disorders support group	Focus group/interview	7	2	In person	RG	13–23
	General practice patient and public group members	Focus group/interview	6	3	Online	JV, JH	72–115
General practice staff and professionals							
	GP, manager/receptionist, practice nurse, voluntary sector provider	Interview	5	5	In person and online	JH, RG	38–143
Total			52	17			

GP, general practitioner.

All interviews and focus groups were audio-recorded after informed consent was provided. We obtained ethical approval for both written and verbal consent processes. Participants gave written or verbal consent as appropriate to the mode of data collection (in person or online) and the participants’ circumstances. Semi-structured topic guides were used. Patient interviews and focus groups concentrated on participants’ experiences of accessing general practice, how their general practice appointment system worked and changed during the pandemic, examples of positive and/or negative experiences and what changes would be desirable. Provider interviews explored participants’ thoughts regarding patient access, their experiences of the realities of provision, current access arrangements, changes because of COVID-19, current and historical access challenges and the influence of policy initiatives on access arrangements (see online supplemental Appendix 1 for the topic guide). No repeat interviews were conducted.

Observations were conducted in seven general practice and Primary Care Network (PCN) premises to understand access arrangements, processes and lived experiences (45 hours across 20 sessions; March 2022–June 2022). Observations typically took place in reception or back-office areas. Informal conversations with various staff provided insights into interactions that were not observed, including team meetings and clinical encounters.

Observations were conducted by JH, RG and LK, usually independently, but jointly for several sessions. The research team was aware of various aspects of the observers’ identities (as researchers with health policy and health communication backgrounds and an academic GP) and reflected on how they affected dynamics in the setting, particularly around the issue of insider/outsider. Contemporaneous hand-written field notes were produced.

### Data analysis

Interview and focus group recordings were transcribed by a professional transcription service and then pseudonymised by the research team. Observation field notes were digitised and pseudonymised by the observing researcher. Digitised field notes and transcripts from interviews and focus groups were stored on a secure research network drive. A rapid qualitative analysis^20 21^ approach was conducted, which involved developing a summary analysis template (or ‘RAP’/Rapid Analysis Procedure sheet), in keeping with established practices using this methodological approach,^21^ and populating this for each source transcript or set of field notes. The template included participant and session details, contextual information and spaces to summarise and reflect on how participants’ experiences mapped to different access dimensions, examples of good/poor ‘fit’ between services and patient needs, COVID-19 impacts on access arrangements and suggestions for improvements. Multiple members of the research team summarised data, including from sources that they generated, and those of others (47% of cases across the data set). This process supported both the development of broad familiarity with the whole data set across the team and also acted as a quality checking measure to ensure that approaches to summarising were accurate to the data source and consistent across the team. The developing templates and summary content were discussed in ongoing team meetings to ensure a consistent process and application. Themes within and across data sources were identified and incorporated into the summaries. The data was then applied abductively to the dimensions of access as human fit, with analysis moving iteratively between empirical data and theoretical framework to develop understanding. Information power^22^ was monitored through ongoing team discussions, and once sufficient data had been gathered to address our research questions, no further data collection was undertaken.

## Results

This research found the realities of access for patients and staff to be very different, made more divergent by specific circumstances following the pandemic including rapid rule changes and insufficient communication. The access as human fit conceptualisation contextualises these distant experiences as a lack of fit across specific dimensions of access, corresponding to the abilities of patients and staff during the journey of care seeking. Three dimensions of access from figure 1—‘Ability to perceive and Ability to approach or be approachable’, ‘Ability to reach and Ability to be available and to accommodate’ and ‘Ability to engage and Ability to be appropriate’—are useful to present data on the gaps we observed between the access experiences of patients and staff. These dimensions are not discrete containers; they should be understood as interactive, overlapping and inter-related concepts, and their interplay is illustrated below.

Extracts from interview and focus group transcripts are denoted with a randomly generated ID, abbreviated data source type and month and year of data collection for example, (A52jd, Int(08/22), Patient). Data relating to observations is denoted with Practice/PCN code, ‘Obs’, and months and year observations took place at that site, for example, (P17, Obs(03-04/22)).

### Ability to perceive and ability to approach or be approachable

‘Ability to perceive’ relates to patients perceiving the need for care and their perceptions about potential engagement with services, mediated by expectations and trust. ‘Ability to approach and be approachable’ includes both staff’s ability to approach patients via outreach and their *approachability*, shaped by information available to patients about services and transparency of care processes.^11^

Approachability of services was affected by general practices’ efforts to control practice space(s) to reduce COVID-19 spread. Associated changes to access arrangements, particularly increased remote consultations and care navigation, were seen by some patients as *exceptional* rather than a direction of travel dramatically accelerated by the pandemic. Some patients appreciated the increased flexibility associated with remote appointments; others found the emphasis on the technology, systems and processes associated with them—particularly telephone and online appointment request tools—reduced approachability (A823k, FG(07/22)).

There was a sense expressed by some patients that they no longer knew the expectations of them as patients, which reflects the concept and theory of candidacy,^23^ and found the array of options for engaging with their practice overwhelming. This created unpredictability about what would happen, often seen through the ‘COVID-19-related changes’ lens, and contrasted with simpler, more predictable arrangements pre-pandemic such as arriving at the practice, taking a numbered ticket and waiting (A2246, FG(06/22)). This connected to a broader narrative that, by mid-2022, the pandemic was being used as an excuse for introducing access and service delivery arrangements which were unsatisfactory for patients but preferable to staff in general practice.

“And they keep blaming it all on COVID which is not an excuse anymore. Because we’ve learned to live with it now, haven’t we? You know, if hospitals can see patients face-to-face now and you’re going in and your seeing doctors in there. Why can’t surgeries start doing the same? You know, face-to-face now. And actually seeing patients. Getting the ball rolling back again as normality has gone back.”(A62EP, FG(07/22), Patient 2)

Some patients noted that systemic challenges (eg, pressures on health services and recruitment issues) were at least in part responsible for current access arrangements (A72mm, FG(07/22); A2246, FG(06/22)), but this should not prevent their needs being recognised by their practices (A72mm, FG(07/22)). A group of mainly older patients from a minority ethnic group, many with limited English proficiency, expressed a belief that their GPs were deliberately restricting their access. Some felt they were perceived as less important because they were older (A2246, FG(06/22)). Other patients thought GPs were primarily working from home (A62EP, FG(07/22)). A practice manager (PM) interviewee speculated that this erroneous perception—and similarly that the practice’s doors were ‘shut’—could be traced to national media messaging framing general practices as closed or that GPs were being lazy or ‘hiding behind their screens’ (A12py, Int(07/22), PM).

This highlighted a gap between patient and staff ‘realities of access’, co-existing and interacting particularly within the dimension of ‘Perceive and Approach’ and mediating the extent to which human fit was realised. Closely related was a sense from staff that the pandemic had fundamentally shifted patient expectations towards a more consumerist–‘Amazon-ified’–mindset where they should be able to access care they want, in their desired mode, virtually immediately (A12py, Int(07/22), PM). At this time, practices were struggling to cope with increased demand, managing staff absence, and recruitment and funding challenges. Some practice and PCN staff did talk about outreach services they had offered (eg, to people unhoused) but noted less ‘bandwidth’ to provide such proactive services or encourage patients to attend screening appointments.

To address some drivers of the realities of the access gap, certain practices made use of a local service that supported and taught individual patients to navigate appointment systems and digital tools. This created a space where patients could find out how to successfully engage with practice systems, and thus increase the approachability of the practice, as well as learn about additional context for why things were working as they were. For instance, a patient who was told that submitting prescription requests online, rather than on paper, freed up staff to do other things like answering the phone, then understood the value of this and switched to doing so (A91zw, Int(05/22)).

### Ability to reach and ability to be available and to accommodate

Patients’ abilities and capacity to ‘Reach’ healthcare shapes their successful engagement with services. This dimension includes factors relating to their personal circumstances such as their availability, transport access, digital literacy and broader factors such as knowledge about the healthcare system and communication skills. For staff, being available and accommodating includes building accessibility through phone and online systems, opening hours, appointment systems and the approach of individuals within the organisation.^11^

We observed increasing variability of access arrangements across practices in terms of the ways patients could reach practices and how accommodating the practices were. While all practices shifted to conducting a significant number of appointments by phone (or rarely, video calls), there was considerable variety in how this was enacted. Practices also varied in when and how they relaxed some COVID-19 protocols. Some practices tried to revert systems and processes back to ‘normal’ as much as possible (A12py, Int(07/22), PM) whereas others either lacked motivation to, felt it was not practically feasible given demand and capacity, or both. Some practices struggled to make confident decisions, particularly around control of common spaces within building(s), in the context of central guidance perceived as ambiguous (P4, Obs(03-05/22)).

Multiple practices primarily operated a ‘first come, first served’ system involving an intense daily start with dozens of patients waiting on the phone and a limited number of appointments. This was problematic for patients’ ability to reach care. Many described having to phone the minute the practice opened, sometimes for multiple consecutive days, to secure an appointment, and one person described the physical challenge of doing this with a condition affecting phone grip (A2246, FG(06/22)).

Some practices offered call-backs for patients queuing on the phone (A23oc, Int(08/22), Clinician), others did not have this facility. Some provided patients a 2-hour time slot for a telephone appointment (eg, A23oc, Int(08/22), Clinician), others told patients they would receive a call at some point that day (A11qm, Int(03/22), Clinician). This was challenging for patients and meant they might not answer because they were not in a sufficiently private space.

Once appointments were all booked, reception staff would often direct patients to call NHS111, visit the Walk-In Centre or A&E or call back the next day. Some practices had policies where patients were requested to complete an online appointment form if, for instance, there were no more telephone appointments left. Some patients reported accessibility issues with such services:

“This new service, where they've got, where you have to fill in a form online. That’s not good for a lot of people. Like my mum is 85 and has got dementia. She hasn't even got; she can't even work a phone. But I have trouble even on a small phone, it’s not always accessible. So how else are you supposed to get an appointment, 'cause that seems to be the only way you get an appointment now, doing it electronically.” (A42n4, Int(07/22), Patient)

Staff sometimes had to support patients lacking internet access or digital literacy by completing their online forms for them–a time-consuming task that occupied phone lines.

For patients, being held on the phone in long queues without confidence they would ultimately receive an appointment was frustrating (A2246, FG(06/22)). Some patients would vent frustration with, and in some cases abuse, reception staff. The emotional toll on staff was clear, and several recounted traumatic stories of abusive patients. One practice manager explained that incidents of aggression and complaints had increased during this period from an average of three per month to as many as six per day (P17, Obs(03-04/22)). Reception staff described strategies to diffuse anger, such as expressing empathy and affirming patients’ expressions of frustration. However, observations revealed this could sometimes be perceived as inauthentic and frustrate patients further. This highlights the bi-directional interconnection between the dimensions of ‘Perceive and Approach’ and ‘Reach and Be Accommodating’. When it is not possible to accommodate a patient’s access request, then approachability aspects, such as openness, empathy and communication skills, are important in shaping the interaction with a patient, but the response can be unpredictable and fuel stress.

Staff highlighted that some patients know what they want from their practice and how to ask for it, whereas others do not (A91zw, Int(05/22), VCSE). One patient talked about knowing how to be ‘devious’ with practice systems to get access (A60jg, Int(12/21), Patient). In one practice, there was confusion about how a small group of patients seemed to consistently get through on the phones and secure appointments (P17, Obs(03-04/22)). Receptionists deemed them less ‘worthy’ than other patients that always seemed to miss out. This suggests the pandemic-driven shift by many practices to greater reliance on remote access may have compounded inequities in terms of different patients’ abilities to reach care.

### Ability to engage and ability to be appropriate

‘Ability to engage’ includes patients’ care utilisation abilities and capacity, shaped by having sufficient information, and their degree of empowerment. For staff, ‘Ability to be appropriate’, factors including coordination, continuity and care quality must be met in ways appropriate to an encounter.

Observing reception staff and patient interactions revealed close connections that culminated in service appropriateness for patients. Reception staff–often with titles such as ‘care coordinators’–were engaged in complex and nuanced ‘care navigation’, that is, understanding patients’ needs, directing them to appropriate services or professionals, while often managing patients’ expectations about their potential experiences. This can be understood as being appropriate. In many instances, a member of the reception staff, knowing the circumstances and health issues of a patient, supported them in navigating the practice and/or broader health and care system effectively. This personal knowledge of the patient–thus relational continuity–was coupled with experience and knowledge of systems and processes enabling staff to understand where rules could be bent or circumvented (P4, Obs(03-05/22)). This work had reportedly increased over the pandemic (P30, Obs(04-06/22)). The extent to which reception staff embodied this understanding was a significant factor determining how far the practice could ‘Be Appropriate’ for individual patients and shape the realm of possible engagement for the patient through providing information. However, some practices had experienced significant reception staff turnover, partly due to demand pressures in general, intermittent workforce illness and increased patient aggression (P8, Obs(03-04/22)). This inhibited the ability of reception staff to do this work and be appropriate. One patient highlighted a perceived lack of appropriate communication from practices to explain why receptionist staff were asking for details that could be considered medically sensitive:

“It was, I think from the point of view of the surgery, they haven’t been able to communicate properly to the patients that yes, they’ve got through the system, they’ve got to a receptionist and people get upset when the receptionists are asking them what they consider to be delicate questions. But that receptionist has been told to ask the questions, so that we can put you in touch with the right physician at this end. That hasn’t been explained properly so some of the people are getting upset because they are having these, they don’t want to ring in because they are getting these intrusive questions.” (A60jg, Int(12/21), Patient)

Relational continuity more typically associated with the patient-clinician dynamic was also an issue. A group of patients with mental health issues emphasised frustrations that the pandemic had affected their ability to speak with a doctor that knew their history and challenges. This diminished continuity and caused distress due to re-telling their stories to different health professionals with different levels of receptivity and understanding (A72mm, FG(07/22)).

Bringing patients together to discuss access appeared to increase their sense of empowerment regarding their ability to engage services. Sharing stories about approaches to obtaining appointments or care often caused others to reflect on their experiences with their own practice and how they might increase their chance of a desirable outcome (A136r, FG(08/22)). There was, however, also a compounding of divergence in ‘access realities’ between patients and staff. The group of older patients from a minority ethnic group, for example, shared experiences of trying to access different practices, and this affirmed narratives around the pandemic being used as an excuse to limit care or for GPs to avoid seeing patients face-to-face (A2246, FG(06/22)).

## Discussion

### Summary

The access as human fit conceptualisation identified important gaps between patient and staff experience mediated by the COVID-19 pandemic. Patient and staff interview data, focus groups with underserved patients and observations within general practice reception areas were presented in relation to three pairs of dimensions of access as human fit. These data highlight the consequences of the profound shock of the pandemic, particularly the evolving adaptations to access-related systems and processes driven by a need to deliver care as safely as possible, and national-level narratives about the pandemic and general practice. This drove a divergence of the access realities inhabited by patients and staff, with knock-on consequences for multiple dimensions of access, thereby pulling the dimensions of access as human fit further apart. Those with existing unmet needs were particularly exposed to this phenomenon, suggesting a compounding of access inequalities. Understanding divergent access realities suggests a need for targeted interventions to improve the human fit by bringing patients and staff together in dialogue and empathy to address broken trust, misunderstandings and the consequences of misinformation.

### Strengths and weaknesses

A key strength is the application of the access as a human fit conceptualisation to the lived experiences of those providing and seeking general practice services in the context of the pandemic. The idea of ‘fit’ has widespread support in research on access,^8^ and the human fit framework has been robustly iterated and developed.^11 12^ The focus on one geographical area is a potential limitation in terms of generalisability, but this should be considered alongside the reality that general practice is both highly diverse across scales and subject to shared forces around the GP contract and societal-level trends. Focusing on one geographical area also permitted a significant depth of engagement to understand the lived realities of access for stakeholders, aspects of which are reported in the literature (see below). Practices and patients were diverse and reported issues and experiences reflecting national-level concerns and rhetoric. While we did not formally explore this, the data appear to show that while our participants came from different backgrounds and interacted with different healthcare organisations, their access needs are universal. The access as human fit conceptualisation may support identification and exploration of these universal needs within and across societal and healthcare contexts. The participatory approach involving the Community-Based Research Team throughout the research is a significant strength and helped to ensure perspectives of a group with diverse characteristics, roles and experiences were reflected.

### Comparison with existing literature

The findings complement other studies of access to general practice during the COVID-19 pandemic.^224^,^26^ The access as human fit application responds to longstanding calls for a more theory-informed approach to assessing access changes within UK general practice, especially regarding health inequalities, which have worsened due to the pandemic.^27^,^29^ This paper adds substance to the understanding that access policy and processes that exclusively focus on timeliness have the potential to drive invisible unmet need and thus compound inequalities.^11 30^ The data also highlights the crucial role reception staff play in mediating human fit, via moral positioning and other means, within primary care settings,^31 32^ and provides further evidence regarding the emotional tolls faced by staff in these roles.^32 33^

Other ongoing research focused on GP access is also relevant to understanding the consequences of the pandemic for systems and process, for example, the GP-SUS (Access to General Practice: Innovation, impact and sustainable change) project being delivered by University of Warwick and University of Oxford,^34^ and THIS Institute’s ‘Improving access to primary care’ project that uses the candidacy framework.^23^ Candidacy provides a valuable framework for exploring the requirements on patients to demonstrate their eligibility to receive care and negotiate systemic challenges; however, it does not engage with the staff ‘side’ of the equation and what happens after people have ‘seen’ themselves as viable candidates for care. By encompassing patient and staff realities, access as human fit can illuminate the interpersonal and situational factors that shape access within a context of variable needs, abilities and capacity at different scales.

### Implications for research, practice and policy

This research adds further evidence to previous critiques of general practice access policy, processes and research which assume and operationalise a narrow definition of access that prioritises speed or appointment quantity over other factors.^1 6 11 30^ Access research should embrace the access as human fit framework, or similar comprehensive frameworks, rather than defaulting to definitions of timeliness or what can be measured, which miss important factors and may drive inequalities. Practices and Primary Care Networks can adopt this view of access within their daily work to realise and address gaps between their service provision and patients’ needs and the gaps between perceptions of patients and staff. The findings presented in this paper directly informed the production of the Optimising Access Through Human Fit (OATH) resource set (www.oath-access.com) as part of the broader project. These resources include vignette activities based on the observations designed to be used by patients and practice staff to understand each other’s experiences and address the gaps revealed in this research.

Crucially, general practice access policy also needs to adopt a broader view of access to stop repeatedly failing to improve access equity. Doing so would make the gaps described in this paper visible, which should create opportunities to appropriately fund general practice to identify those in their populations with unmet needs and address them in a proactive, holistic way. Crucially, investment is necessary to help general practice overcome the loss of trust that is reflected in the gaps in understanding described in this paper. Finally, adopting a view of access as human fit allows for the health service to be seen as a service of people whose ability to provide appropriate care is affected by system factors such as workforce shortages and lack of funding. Investment to improve relations between patients and practices is key to the sustainability of the workforce and thus essential to the provision of equitable care.

## Supplementary material

10.1136/bmjopen-2024-095120 online supplemental file 1

## Data Availability

No data are available.
