# Supplementary material for: Understanding the human fit of access during initial recovery from the COVID-19 pandemic: a qualitative study in English general practice
Source: BMJ Open. 2025 Oct 15;15(10):e095120. doi: 10.1136/bmjopen-2024-095120 (PMC12530364; doi:10.1136/bmjopen-2024-095120)
Supplement: online supplemental file 1 [file bmjopen-15-10-s001.docx]

**Appendix 1: Interview and focus group topic guide**

Patients

Was is the experience of trying to access care from your GP surgery like? How does the appointment system work?

Have you had any specific experiences in the past that stand out as good (or someone you are close to)?

What was it about the experience that made them stand out as positive?

Have you had any specific experiences in the past that stand out as bad (or someone you are close to)?

What was it about the experience that made them stand out as negative?

Would you like to see any particular changes to the way accessing care through your practice is organised?

General practice staff and professionals

What does patient access mean to you? How do you think about the issue? Is there any difference between your personal conceptualisation of access and how it is treated as an organisation?

What is your current approach to organising patient access in your practice?

What changes resulted from Covid-19 and what was already in place before that?

What have been your historical access challenges pre-Covid-19?

How have policy initiatives shaped your practice systems re access now and in the past?

What does patient access to general practice mean to you? How do you think about the issue?

What are some access issues specific to the local community or sections of it here?

How does your work relate to patient access?
