# Supplementary material for: Understanding the human fit of access during initial recovery from the COVID-19 pandemic: a qualitative study in English general practice
Source: BMJ Open. 2025 Oct 15;15(10):e095120. doi: 10.1136/bmjopen-2024-095120 (PMC12530364; doi:10.1136/bmjopen-2024-095120)
Supplement: Supplementary file 2 [file bmjopen-2024-095120.reviewer_comments.pdf]

PEER REVIEW HISTORY

BMJ Open publishes all reviews undertaken for accepted manuscripts. Reviewers are asked to complete a checklist review form and are provided with free text boxes to elaborate on their assessment. These free text comments are reproduced below.

ARTICLE DETAILS

Title (Provisional)

Understanding the human fit of access during initial recovery from the COVID-19 pandemic: a qualitative study in English general practice

Authors

Hammond, Jonathan; Goulding, Rebecca; Drinkwater, Jessica; Kent, Lindsey; Bailey, Simon; Checkland, Kath; Voorhees, Jennifer

---

VERSION 1 - REVIEW

**Reviewer**	**1**
**Name**	**Lavergne, M.**
**Affiliation**	**Dalhousie University Faculty of Medicine, Department of Family Medicine**
**Date**	**08-Feb-2025**
**COI**	**None**

---

bmjopen-2024-095120

I appreciated the opportunity to review this paper. Its central contribution of drawing attention to the different realities of access for patients and staff is important, and the inclusion of varied perspectives is a key strength. I hope comments below are helpful in revising this manuscript.

Abstract

The analysis is described as abductive within the abstract, but I noticed this isn't defined or explained within the text.

Introduction

1. This analysis, and the cited paper by Voorhees et al build constructively on Levesque to highlight that access occurs at the interface between people (patients and staff), and the corresponding importance of the human fit between patients' needs and the ability and capacity of people in the health workforce. That said, the "Access as Human Fit" ladder is substantially simplified – how this was developed, and it's intended contribution beyond existing excellent work Voorhees et al did to extend Levesque et al. wasn't clear to me.

To explain further, this stood out in factors such as “affordability” and “appropriateness” being framed as staff “being affordable” or “being appropriate. This frames affordability and appropriateness as a property of people, and systems are only broadly represented. Simplification also loses other important detail. For example, it does not attend to the various forms of costs people experience (which may not be determined by or within the control of people providing care). This is a minor point, but I found it initially challenging to orient myself to the ladder diagram, as movement is framed as being down rather than up.

I would have found more discussion of the development of the ladder and the choices made to simplify previous work to be helpful as this is so central to subsequent analysis. Alternatively, I feel the introduction could constructively cite Levesque and Voorhees, and focus more on current context for how access is measured, defined, and incentivized in more limited ways. This seems key in motivating this work but is discussed only to a limited extent.

2. The aim of this paper is described as exploring the impact of the pandemic on general practice access in the English NHS, from the perspectives of patients and staff. Authors then note that the wider project also coproduced resources to support improvements to general practice access processes and provide national policymakers with evidence to inform policy. I felt that this framing confused the paper. Methods and approaches to produce resources are distinct from the analysis of general practice access. And both are not comprehensively explained in the paper (and likely cannot be within word limits).

Methods

3. Analysis is described as “rapid qualitative analysis” with a “summary analysis template” used for each transcript or set of field notes. I would have found more detail on what informed this template and what was contained within it very helpful. Whether and how the development of themes connected to the template wasn’t clear to me. Similarly, authors write that the data was applied to dimensions of access as human fit. How or if the template reflects these dimensions would be helpful to know.

Results

4. The data presented focuses on three dimensions of access. It’s explained that this is because they were most striking in terms of the distance between the access experiences of patients and staff. That left me wondering about other dimensions where we might learn from what the presence of fit looks like. This could be briefly explained.

5. The methods mention multiple patient groups. The results mention a group of older patients from a minority ethnic group, but I was curious how perspectives varied among other patient groups. I was also unsure how analysis incorporated these varied perspectives.

Discussion

6. In reading through the results, it was ultimately not clear to me how framing in terms of “access as human fit” depicted as a ladder as opposed to simply referencing Voorhees and Levesque materially changed discussion. I think the unique contribution of this framing

should be explained more carefully, particularly given that adopting this image and associated resources is put forth as a main implication of this research.

7. I particularly appreciated the inclusion of varied perspectives, including reception staff. That reception staff may be required to mediate fit is a very important observation. One of my discomforts with the framing of “Be Accommodating” or “Be Appropriate” is variation in supports that enable this, actual capacity to accommodate/deliver quality care, and as well as in expectations for what is accommodating and appropriate (which may vary across lines of power, including role, as well as potentially gender, racialization, social class etc). I think there is an opportunity to further explore this in the discussion.

8. Authors write “Understanding divergent access realities suggests a need for targeted interventions to improve the human fit, by bringing patients and staff together in dialogue and empathy to address broken trust, misunderstandings, and the consequences of misinformation.” While clearly this is supported by results, I wonder about how this might be enabled within an overstretched system, and what fundamental gaps in resources and capacity should also be considered.

Conclusion

9. The conclusion links to a set of resources. Given that their development isn’t the focus of this research and they are not described within the manuscript this didn’t seem to follow clearly from the results presented.

**Reviewer**	**2**
**Name**	**Blome, Christine**
**Affiliation**	**University Medical Center Hamburg-Eppendorf , Health Services Research in Dermatology**
**Date**	**28-May-2025**
**COI**	**None**

This is a paper that examines access to care during the Covid-19 pandemic, using the human fit as access model. It is very well written, combines multiple qualitative methods and multiple stakeholder persepctives, and describes relevant and interesting findings.

I have a few comments and suggestions, especially regarding the Methods section. It would be important to complete the COREQ checklist and/or mention the relevant content in the text, e.g., the qualifications of those who conducted the interviews. It would also be good to provide the interview guides as an appendix.

Participants characteristics should be presented in a table—for example, which conditions they had.

It would be helpful to specify the duration of the interviews and focus groups and whether these took place online or in person.

Another open question concerns the sample size and the justification for its extent.

The methods of the qualitative analysis are, in my view, described too superficially, as the cited reference 17 does not provide a more detailed approach to a rapid qualitative analysis but only touches on it very briefly.

It is stated that multiple summaries were cross-checked. How many exactly?

There seems to be a contradiction between the data collection period, which ended in June 2023, and the paper's title referring to "recovery from the pandemic." The public health emergency of international concern was only declared over by the WHO in May 2023. (The pandemic itself actually has not yet been declared over.) Thus, the pandemic was still ongoing during the vast majority of the data collection period. Related to this, I was surprised that the results do not seen to show any views that that certain precautionary measures might still have been justified.

As a minor point: The framework is sometimes called "access as human fit" and sometimes "access of human fit." Which is the correct version?

Overall, this is a relevant, methodologically well-conducted, and theoretically well-grounded paper.

VERSION 1 - AUTHOR RESPONSE

Comment code	Comment	Response
R1	I appreciated the opportunity to review this paper. Its central contribution of drawing attention to the different realities of access for patients and staff is important, and the inclusion of varied perspectives is a key strength. I hope comments below are helpful in revising this manuscript.	We appreciate this positive assessment and are grateful for the constructive comments from both reviewers, which have addressed in detail below.
R1.A	The analysis is described as abductive within the abstract, but I noticed this isn't defined or explained within the text.	In the data analysis section, we have added detail on page 5 to explain that the abductive approach involved analysis moving iteratively between empirical data and the theoretical framework.
R1.1	This analysis, and the cited paper by Voorhees et al build constructively on Levesque to highlight that access occurs at the interface between people (patients and staff), and the	Thank you for this helpful steer. Upon reflection we recognise that it is not possible for us to adequately

	corresponding importance of the human fit between patients' needs and the ability and capacity of people in the health workforce. That said, the "Access as Human Fit" ladder is substantially simplified – how this was developed, and it's intended contribution beyond existing excellent work Voorhees et al did to extend Levesque et al. wasn't clear to me.<br><br>To explain further, this stood out in factors such as "affordability" and "appropriateness" being framed as staff "being affordable" or "being appropriate. This frames affordability and appropriateness as a property of people, and systems are only broadly represented. Simplification also loses other important detail. For example, it does not attend to the various forms of costs people experience (which may not be determined by or within the control of people providing care). This is a minor point, but I found it initially challenging to orient myself to the ladder diagram, as movement is framed as being down rather than up.<br><br>I would have found more discussion of the development of the ladder and the choices made to simplify previous work to be helpful as this is so central to subsequent analysis. Alternatively, I feel the introduction could constructively cite Levesque and Voorhees, and focus more on current context for how access is measured, defined, and incentivized in more limited ways. This seems key in motivating this work but is discussed only to a limited extent.	explain the process of analysis and distillation that resulted in the access as human fit ladder in this paper, and consequently we have decided to use Voorhees' published adaptation of the Levesque model and leave a more developed consideration of the ladder to a subsequent paper. Therefore, Figure 1 has been updated and references to the ladder have been removed throughout.<br>We have also now edited the introduction to emphasise the important contrast between the policy definitions of access and the more nuanced understanding of access in the academic literature, since this difference is central to the relevance of the need for this work to influence policy.
R1.2	The aim of this paper is described as exploring the impact of the pandemic on general practice access in the English NHS, from the perspectives of patients and staff. Authors then note that the wider project also coproduced resources to support improvements to general practice access processes and provide national policymakers with evidence to inform policy. I felt that this framing confused the paper. Methods and	We recognise that this framing could be clearer. We have removed reference to the OATH resource set from the methods section entirely. We have amended the conclusion to include reference to the resource set, which the data explored in this paper directly

	approaches to produce resources are distinct from the analysis of general practice access. And both are not comprehensively explained in the paper (and likely cannot be within word limits).	informed, as part of the broader project for context and because it is relevant to the implications for policy and practice.
R1.3	Analysis is described as “rapid qualitative analysis” with a “summary analysis template” used for each transcript or set of field notes. I would have found more detail on what informed this template and what was contained within it very helpful. Whether and how the development of themes connected to the template wasn’t clear to me. Similarly, authors write that the data was applied to dimensions of access as human fit. How or if the template reflects these dimensions would be helpful to know.	We have included additional detail regarding the development and content of the template, including its relationship to the dimensions of access as human fit on page 5.
R1.4	The data presented focuses on three dimensions of access. It’s explained that this is because they were most striking in terms of the distance between the access experiences of patients and staff. That left me wondering about other dimensions where we might learn from what the presence of fit looks like. This could be briefly explained.	We recognise our wording about these three dimensions being the most striking was imprecise and did not convey what we intended. On p5, we have changed this to say that these three dimensions are ‘useful to present data on gaps that we observed between the access experiences of patients and staff’. The included dimensions relate to a significant proportion of the data we collected and were included to show how the human fit conceptualisation could help us organise and better understand all of this. The other dimensions did not provide examples of fit – these also reflected gaps – however, the data was less extensive for these dimensions and so we chose to focus where it was most concentrated for this relatively brief paper.

R1.5	The methods mention multiple patient groups. The results mention a group of older patients from a minority ethnic group, but I was curious how perspectives varied among other patient groups. I was also unsure how analysis incorporated these varied perspectives.	We analysed data from all patient groups collectively rather than conducting separate analyses by group. Notably, despite recruiting from diverse communities via local groups, we found consistency in core themes around access challenges and preferences. While specific examples and contexts varied between groups, the fundamental issues with access 'fit' were consistent across participant types. We present findings thematically rather than by demographic group because of this.
R1.6	In reading through the results, it was ultimately not clear to me how framing in terms of "access as human fit" depicted as a ladder as opposed to simply referencing Voorhees and Levesque materially changed discussion. I think the unique contribution of this framing should be explained more carefully, particularly given that adopting this image and associated resources is put forth as a main implication of this research.	As noted above, to make the paper clearer overall we have removed the ladder diagram from the paper and used Voorhees' already published figure throughout instead. (For context, the ladder diagram which is used in the resource set is an effort to increase the accessibility of the diagram for a broader audience rather than a replacement for the published diagram. We developed this alongside a version of the ladder that retains and extends the detail further.)
R1.7	I particularly appreciated the inclusion of varied perspectives, including reception staff. That reception staff may be required to mediate fit is a very important observation. One of my discomforts with the framing of "Be Accommodating" or "Be Appropriate" is variation in supports that enable this, actual capacity to accommodate/deliver quality care, and as well as in expectations for what is accommodating and appropriate (which may	This is a helpful reflection, and we agree that the boundary between the individual and the systemic in terms of forces, responsibilities etc is important and deserving of proper attention and discussion. Given that we have reverted to the

	vary across lines of power, including role, as well as potentially gender, racialization, social class etc). I think there is an opportunity to further explore this in the discussion.	published Voorhees' access as human fit figure (and therefore the wording that may conflate individual and system in the ladder is not included) we intend to leave consideration of this issue to a paper currently in development that focuses specifically on the role of receptionists in access where this will be a key element.
R1.8	Authors write "Understanding divergent access realities suggests a need for targeted interventions to improve the human fit, by bringing patients and staff together in dialogue and empathy to address broken trust, misunderstandings, and the consequences of misinformation." While clearly this is supported by results, I wonder about how this might be enabled within an overstretched system, and what fundamental gaps in resources and capacity should also be considered.	We recognise there are fundamental practical challenges in these recommendations and have addressed this further in the final section of the conclusion.
R1.9	The conclusion links to a set of resources. Given that their development isn't the focus of this research and they are not described within the manuscript this didn't seem to follow clearly from the results presented.	As noted in R1.2, we have amended the reference to the resource set in the conclusion to make it clearer that the findings presented also informed the development of a resource set that has direct implications for policy and practice.
R2	This is a paper that examines access to care during the Covid-19 pandemic, using the human fit as access model. It is very well written, combines multiple qualitative methods and multiple stakeholder perspectives, and describes relevant and interesting findings.	Thank you for this positive and encouraging assessment of the paper overall.
R2.1	It would be important to complete the COREQ checklist and/or mention the relevant content in the text, e.g., the qualifications of those who conducted the interviews. It would also	We have added additional detail relating to the relevant elements of the COREQ checklist to the methods section as

	be good to provide the interview guides as an appendix.	suggested, and particularly in Table 1. We have added the researchers involved in data collection and some information regarding positionality and expertise but not included all individual qualifications due to word limit constraints. We have also included the interview and focus group guides as an appendix. We believe these additions provide the transparency needed for readers to assess the rigor and credibility of our qualitative approach.
R2.2	Participants characteristics should be presented in a table—for example, which conditions they had.	Our study design prioritised recruiting typically under-served populations via existing community groups in the geographical areas of the research. We collected minimal demographic data from group participants in order to increase the acceptability of research involvement to them. We have now included Table 1, however, which summarises the types of community groups participants were recruited from and the number of participants per group.
R2.3	It would be helpful to specify the duration of the interviews and focus groups and whether these took place online or in person.	The duration range of focus groups and interviews, and mode of data collection, are now included in Table 1.
R2.4	Another open question concerns the sample size and the justification for its extent.	Our sample size was determined by the research aims and the achievement of sufficient information power (see p5). The 52 participants across 17 sessions – alongside the 45 hours of general practice

		observations – provided rich, contextual data that allowed us to explore the complexity of access experiences during the pandemic period. Additional recruitment ceased when sufficient information had been gathered to address our research questions given our theoretical framework and purposive sampling approach.
R2.5	The methods of the qualitative analysis are, in my view, described too superficially, as the cited reference 17 does not provide a more detailed approach to a rapid qualitative analysis but only touches on it very briefly.	We have strengthened this aspect of the methods with an additional reference and clarification of what a rapid qualitative analysis entailed, in this case.
R2.6	It is stated that multiple summaries were cross-checked. How many exactly?	Thank you for highlighting this lack of detail. We have deepened the explanation of the analysis process on p5 to be clearer about what we mean: "Multiple members of the research team summarised data, including from sources that they generated, and those of others (47% of cases across the data set). This process supported both the development of broad familiarity with the whole data set across the team but also acted as a quality checking measure to ensure that approaches to summarising were accurate to the data source and consistent across the team."
R2.7	There seems to be a contradiction between the data collection period, which ended in June 2023, and the paper's title referring to "recovery from the pandemic." The public health emergency of international concern was only declared over by the WHO in May	Thank you for pointing this out. This work was funded by a call that focused on recovery from the pandemic, which came out in summer 2020. We agree that the

	2023. (The pandemic itself actually has not yet been declared over.) Thus, the pandemic was still ongoing during the vast majority of the data collection period. Related to this, I was surprised that the results do not seen to show any views that that certain precautionary measures might still have been justified.	recovery is a long road, and is far from over even still. We have now changed the title to reflect this was recovery from the initial phase of the pandemic. We have also clarified the dates of data collection relevant to this paper, given the separation of the resource development from the findings of this paper. Data presented is from Dec 2021 to Aug 2022. As we note in the 'Study design and setting' section of the Methods, this was a point where there was a significant shift in national regulations and political and media sentiment around the pandemic, which included removing 'plan B' measures, the duty to self-isolate, social distancing, and the issuing of Living with COVID-19 guidance. There was a strong sentiment amongst patient participants that, at that time, it was important to move on from the pandemic and 'get back to normal' and 'stop using it as an excuse.' This contributed to a gap in the realities of health care workers and patients in terms of how they perceived the reality of the pandemic at that stage. The reference in the title to recovery from the pandemic speaks to this period where attempts were made to shed many of the rules and services (such as free testing) that had come to

		characterise the pandemic before that point.
R2.8	As a minor point: The framework is sometimes called “access as human fit” and sometimes “access of human fit.” Which is the correct version?<br>Overall, this is a relevant, methodologically well-conducted, and theoretically well-grounded paper.	‘Of’ was a typo. Now corrected.<br><br>Thank you!

VERSION 2 - REVIEW

Reviewer **1**

Name **Lavergne, M.**

Affiliation **Dalhousie University Faculty of Medicine, Department of Family Medicine**

Date **19-Aug-2025**

COI

Thank you for the opportunity to review this manuscript. I feel the choices made have focused the paper and improved clarity. I just have a couple additional comments related to changes made.

Introduction: Authors have added the text “Despite historically simplistic policy definitions of access” and a relevant reference. I would have found it helpful to briefly describe these definitions, particularly for international readers who might not be familiar with the policy context.

Discussion: I felt policy implications could be further developed. Authors note that constrained resources that make improving access challenging are a policy choice. I would have found more specific policy implications or details on these resources helpful, here also as someone not familiar with the policy context.

VERSION 2 - AUTHOR RESPONSE

Reviewer 1 comments:
R1.1: Introduction: Authors have added the text “Despite historically simplistic policy definitions of access” and a relevant reference. I would have found it helpful to briefly describe these definitions, particularly for international readers who might not be familiar with the policy context.
We have clarified that these definitions of access in England focus on timeliness.

R1.2: Discussion: I felt policy implications could be further developed. Authors note that constrained resources that make improving access challenging are a policy choice. I would have found more specific policy implications or details on these resources helpful, here also as someone not familiar with the policy context.
We have reworked the final paragraph to further develop the policy implications in line with the Reviewer's helpful steer.
